# Larval Metabolic and Physiological Mechanisms Underlying Resistance to Chinese Sacbrood Virus in *Apis cerana*

**DOI:** 10.3390/insects16121283

**Published:** 2025-12-18

**Authors:** Yang Lü, Liyuan Zheng, Wenyao Ouyang, Aqai Kalan Hassanyar, Songkun Su, Zhiguo Li

**Affiliations:** 1 College of Animal Sciences, College of Bee Science, Fujian Agriculture and Forestry University, Fuzhou 350000, China; ly89617974@outlook.com (Y.L.); z18065828890@163.com (L.Z.); oyooooung@163.com (W.O.); hassanyar400@gmail.com (A.K.H.); 2 Heilongjiang Academy of Agricultural Sciences, Mudanjiang 157000, China; 3 Department of Animal Sciences, Faculty of Agriculture, Alberoni University, Kapisa 1254, Afghanistan

**Keywords:** *Apis cerana*, chinese sacbrood virus, disease resistance, metabolomics, antioxidant enzymes

## Abstract

CSBV is a major pathogen threatening the health of *A. cerana*, but the mechanisms underlying natural resistance in its larval host remain poorly understood. To elucidate this, we established resistant (R) and susceptible (S) larval phenotypes through controlled CSBV inoculation and combined untargeted metabolomics with biochemical assays. Metabolomic profiling revealed a markedly disrupted metabolic state in S larvae, with 8272 differential metabolites compared to controls, versus a more moderate response in R larvae 4208 metabolites. Key pathways, including galactose and glycerophospholipid metabolism, were significantly perturbed. Crucially, R larvae exhibited a profoundly enhanced antioxidant defense in the gut, with activities of catalase, glutathione S-transferase, and superoxide dismutase being 2.5-fold, 1.5-fold, and 2.0-fold higher, respectively, than in S larvae. Our findings demonstrate that CSBV resistance is linked to a modulated metabolic response and a robust, activated antioxidant system, providing new insights into host–pathogen interactions and potential strategies for safeguarding pollinator health.

## Simple Summary

Chinese Sacbrood Virus (CSBV) is a major threat to honeybee populations. In this study, we infected *Apis cerana cerana* (*A. c. cerana*) larvae with CSBV and identified two distinct groups: resistant larvae that survived and developed normally, and susceptible larvae that showed disease symptoms and died. We discovered that resistant larvae have different metabolic patterns and stronger activity of key antioxidant enzymes in their gut compared to susceptible larvae. This enhanced antioxidant defense appears to be a crucial factor in their ability to resist the viral infection. These findings help us understand why some honeybees can survive CSBV infection and may contribute to developing strategies for protecting pollinator health.

## 1. Introduction

The Asian honey bee, *A. cerana*, is a native and crucial pollinating insect in Asia, playing an irreplaceable role in maintaining the balance of natural ecosystems and ensuring agricultural productivity [1,2]. Compared to the Western honey bee (*Apis mellifera*), *A. cerana* demonstrates higher pollination efficiency for native plants and is better adapted to complex mountainous environments [3]. However, in recent decades, due to multiple stressors including habitat destruction, pesticide overuse, climate change, and pathogen spillover, both wild populations and managed colonies of *A. cerana* have drastically declined [2,4]. It is now listed as an endangered species, and its survival status has garnered widespread concern [5].

Among various pathogenic factors, CSBV is the most devastating pathogen for *A. cerana* [6,7]. CSBV is a highly virulent geographic strain of Sacbrood Virus (SBV) [8], belonging to the family Iflaviridae and characterized by a positive-sense, single-stranded RNA genome [9,10,11,12]. This virus primarily infects 3- to 5-day-old larvae, inhibiting the molting and pupation process [13,14,15]. This leads to the accumulation of fluid, forming a typical “sac-like” structure within the larvae [16], resulting in death and potentially the collapse of entire colonies within weeks under severe conditions [17]. Since its initial outbreak in China in the 1970s, CSBV has caused multiple large-scale epidemics, resulting in sustained and significant economic losses to the Chinese beekeeping industry [5].

Honey bees primarily rely on their innate immune system to defend against pathogen invasion [18,19,20]. This system comprises cellular immunity (e.g., phagocytosis, melanization) mediated by hemolymph and humoral immunity regulated by various signaling pathways [21]. Among these, the Toll pathway primarily responds to Gram-positive bacteria and fungi, the IMD pathway to Gram-negative bacteria, and the JAK/STAT pathway plays a key role in antiviral responses. Additionally, RNA interference (RNAi) is a core intracellular antiviral mechanism in bees [22]. It is important to note that the activation of immune responses, particularly melanization, is accompanied by the generation of substantial Reactive Oxygen Species (ROS) [23]. Therefore, an efficient antioxidant system including enzymes such as superoxide dismutase (SOD) and catalase (CAT) is crucial for maintaining immune homeostasis and preventing oxidative tissue damage [24,25]. The insect gut is a critical defensive organ; as the primary interface exposed to the external environment, it constitutes the first line of defense against invading pathogens and toxins [26]. The integrity of gut tissue, its microbial homeostasis, and local immune status directly determine the overall health of the honey bee [27,28].

Viral infections often trigger metabolic reprogramming in host cells and can disrupt immune balance and redox homeostasis. Consequently, the specific alterations CSBV infection induces in the gut metabolite profile of *A. cerana*, the corresponding responses of key immune pathways within the gut, and the potential compromise of the gut’s antioxidant defense system remain to be elucidated. Metabolomics, capable of unbiasedly revealing the overall changes in all small molecule metabolites of an organism under pathological states, serves as a bridge connecting gene expression and the final phenotype [29,30,31].

In this study, we systematically investigated the pathogenic mechanisms of CSBV in *A. cerana* larvae by analyzing its impact on larval gut metabolites, and antioxidant enzyme activities following controlled inoculation. Our findings provide crucial scientific insights into CSBV pathogenesis and establish a foundation for developing effective strategies to control this devastating disease.

## 2. Materials and Methods

### 2.1. Larvae Rearing In Vitro

Larvae of *A. cerana cerana* were obtained from the experimental apiary of Fujian Agriculture and Forestry University. To collect same-age second-instar larvae, a queen was confined to a clean comb within a cage for 24 h for egg-laying. The comb was maintained in the hive for an additional 96 h before being transported to the laboratory for grafting. Larvae were reared in vitro using a 48-well cell culture plate. The larval diet was prepared according to Hassanyar et al. [32], consisting of 6 g D-glucose, 6 g D-fructose, 1 g yeast extract, and 50 g royal jelly in 37 mL double-distilled water. The components were mixed thoroughly, pureed, and administered to the larvae for three days, with fresh a diet prepared using the same method for all experiments. Larvae were maintained in an incubator at 34 °C and >90% relative humidity.

### 2.2. Viral Inoculation

The CSBV inoculum was obtained from the strain isolated and purified by Hassanyar et al. [32]. The virus stock solution had a concentration of 1 × 10^6^ genome copies per µL, as determined by quantitative RT-PCR. Larvae of *A. cerana* were divided into experimental and control groups, each group consisted of three replicate cohorts, with each cohort containing 480 larvae, resulting in a total sample size of *n* = 1440 per group. Each larva in the experimental group received a single oral inoculation of 2 µL CSBV solution (total viral dose: 2 × 10^6^ genome copies) mixed into 10 µL of artificial diet. Control larvae were treated similarly but received an equal volume of sterile PBS in place of the virus solution. This feeding was administered once to all larvae following grafting. An uneaten diet was removed after 24 h. Upon defecation, each larva was gently cleaned with sterile tissue and transferred using soft forceps to a new 48-well plate lined with two sterilized filter papers. Larval mortality was recorded daily, and morphological characteristics were examined under a stereo microscope.

### 2.3. Identification of Resistant and Susceptible Larvae

On day 7 post-inoculation, the CSBV-infected larvae were assessed for phenotypic outcomes to categorize them into resistant or susceptible groups. The classification was based on clear morphological signs. Larvae that displayed a progression of symptoms from whitish to yellow, followed by darkening and desiccation, and ultimately failed to pupate, were classified as susceptible (S). Conversely, larvae that did not exhibit these symptoms and successfully developed into the pupal stage were classified as resistant (R).

### 2.4. Integrated Analysis of Prevalence and Viral Load

To integrate the prevalence data with viral load measurements, we performed the following analysis. First, qPCR screening of the control cohort (*n* = 48) revealed a covert infection prevalence of 83% (40/48). These 40 qPCR-positive individuals were then defined as the Natural Infection group for subsequent comparative analysis. The viral loads of this Natural Infection group (NI) were statistically compared with those of the experimentally challenged Resistant (R) and Susceptible (S) groups using a one-way ANOVA followed by Tukey’s post hoc test.

### 2.5. DNA Extraction and SNP Validation

Genomic DNA was extracted from 12 S and 12 R honey bee larvae using the Lab Serv Universal DNA Kit (Thermo Scientific, Waltham, MA, USA) on a KingFisher Flex system. Each larval sample was homogenized in 100 μL of Lysis Buffer until a slurry formed. Then, 5 μL of proteinase K (20 μg/μL) was added, and the mixture was homogenized using a high-throughput tissue grinder (30 s, 40 Hz, repeated 2–3 times), followed by incubation at 65 °C for 20 min. Subsequent steps were automated on the KingFisher Flex system. DNA concentration was measured using a NanoDrop ND-2000 (Thermo Scientific, Wilmington, MA, USA), and integrity was verified by agarose gel electrophoresis. All DNA samples were stored at −20 °C. The candidate SNP, KZ288479.1_95621 targeted PCR amplification followed by Sanger sequencing was performed for all 24 DNA samples. Primer sequences flanking SNP locus were adopted from Hassanyar et al. [32]. PCR reactions were carried out in 25 μL volumes containing 500 ng of genomic DNA, 0.4 μM of each primer, and 12.5 μL of 2 × EasyTaqBuffer (Vazyme, Nanjing, China). The thermal cycling conditions were: initial denaturation at 95 °C for 5 min; 35 cycles of 95 °C for 30 s, annealing at 60 °C for 30 s, and extension at 72 °C for 45 s; with a final extension at 72 °C for 7 min. PCR products were purified and sequenced bidirectionally (Sunya Biotech, Fuzhou, China). Sequence chromatograms were analyzed and aligned to the reference genome using SnapGene software (v 8.2.1) to confirm the genotypes at the target SNP positions.

### 2.6. PCR Amplification, Electrophoresis and Sequencing

A pair of specific primers, yielding a 157-bp product from a previously published sequence [33], were used for PCR amplification. The PCR products were separated on a 1.5% agarose gel and visualized under UV light. Target bands were excised from the gel, purified, and sent for Sanger sequencing to confirm the identity of the amplicon.

### 2.7. RNA Extraction and cDNA Synthesis

Total RNA was extracted from whole honey bee larvae using a commercial RNA kit (Promega, Madison, WI, USA). The extracted RNA was treated with DNase I to remove genomic DNA contamination. RNA concentration and purity were assessed using a NanoDrop ND-2000 spectrophotometer (Thermo Scientific, Wilmington, MA, USA). First-strand cDNA was synthesized from 1 μg of total RNA using a Reverse Transcription Kit (Vazyme, Nanjing, China) with oligo (dT) and random hexamer primers.

### 2.8. Quantitative PCR (qPCR) and Relative Quantification

Quantitative PCR was performed in triplicate using a SYBR Green protocol (Vazyme, Nanjing, China) on a real-time PCR system. The same 157-bp primer set was used for amplification. The actin gene was used as an internal control to normalize the expression levels across all samples. The relative viral expression levels in the S group compared to the R group were calculated using the comparative 2^−ΔΔCt^ method.

### 2.9. Larval Gut Metabolomics Analysis

For the metabolomic profiling of the larval gut, midguts were aseptically dissected from 7-day-old *A. cerana* larvae under stereo microscope on sterilized glass slides using ethanol-sterilized forceps; each gut was carefully extruded by gently pulling the abdomen after securing the head, rinsed in ice-cold PBS, and pooled into samples each comprising eight midguts, with six biological replicates per group—control (CK), R, and S. After weighing to approximately 25 mg, samples were snap-frozen in liquid nitrogen, stored at −80 °C, and shipped on dry ice to Shanghai BioTree Company (Shanghai, China) for metabolomic determination. Metabolites were extracted by low-temperature homogenization with an extraction solution containing isotope-labeled internal standards, followed by ultrasonication in an ice-water bath and centrifugation at 13,900× *g* for 15 min. The supernatant was analyzed using a Vanquish UHPLC system (Thermo Fisher Scientific, Bremen, Germany) with the following chromatographic conditions: mobile phase A: 25 mM ammonium acetate and 25 mM ammonia water, and phase B: acetonitrile, both maintained at 4 °C with an injection volume of 2 µL. Data processing and metabolite identification were performed using a custom R package (v 4.4.1) referencing the BiotreeDB (V3.0) database, and in-house R tools were used for visualization.

### 2.10. Determination of Gut Antioxidant Enzyme Activities

The specific activities of the antioxidant enzymes catalase (CAT), glutathione S-transferase (GST), and superoxide dismutase (SOD) were assayed following established protocols from our previous studies [34,35,36]. Total protein was extracted from each pooled gut sample using a commercial extraction kit (Mingke Biotechnology, Beijing, China) according to the manufacturer’s instructions. Briefly, six independent replicates, each consisting of a 25 mg larval intestinal sample, were processed. The samples were homogenized in extraction buffer, incubated on ice for 30 min, and centrifuged at 14,000× *g* for 10 min at 4 °C to collect the supernatant. The protein concentration of each extract was quantified using a bicinchoninic acid (BCA) assay. Enzyme activities were determined using double-antibody sandwich enzyme-linked immunosorbent assay ELISA kits (mlbio, Shanghai, China). For each assay, 50 µg of total protein from each sample or standard was added to the antibody-pre-coated wells, followed by the addition of a horseradish peroxidase (HRP)-labeled detection antibody. After incubation and washing, the color reaction was developed with tetramethylbenzidine (TMB) substrate, and the absorbance was measured at 450 nm. The activity of each antioxidant enzyme was calculated based on its respective standard curve and expressed as units per milligram of total protein (U/mg).

### 2.11. Statistical Analyses

All statistical analyses were performed using GraphPad Prism version 9.0. Survival curves for the CSBV-inoculated and PBS control groups were plotted using the Kaplan–Meier method and compared with the log-rank (Mantel–Cox) test. For comparisons between two groups of continuous data, an unpaired Student’s *t*-test was used. For comparisons among three or more groups, statistical significance was determined by one-way analysis of variance (ANOVA) followed by Tukey’s post hoc test. Data are presented as the mean ± standard deviation (SD) for normally distributed datasets, though the standard error of the mean (SEM) is used in graphical representations to illustrate the precision of the mean estimate. Differences were considered statistically significant at *p* < 0.05.

## 3. Results

### 3.1. CSBV Inoculation in A.c. cerana Larvae and Screening of Resistant and Susceptible Larvae

**Figure 1:**
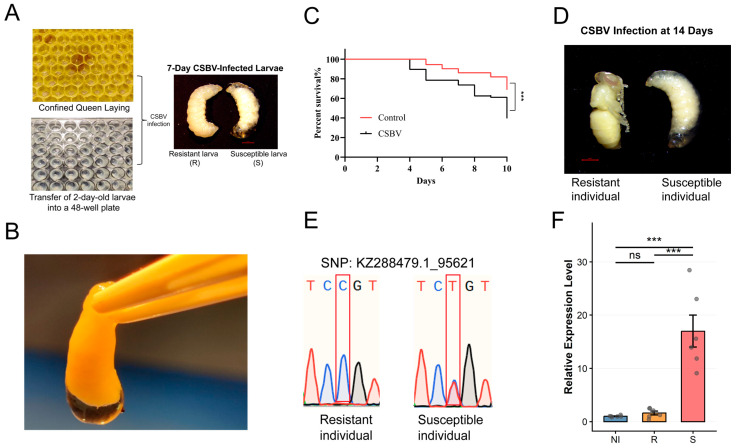
CSBV inoculation and selection of resistant and susceptible larvae. (**A**) Rearing of *A. cerana* larvae and the CSBV inoculation procedure. Resistant and susceptible larvae were distinguished at 7 days of age. (**B**) Typical morphological characteristics of CSBV-infected larvae. (**C**) Kaplan–Meier survival curves of larvae following CSBV inoculation versus control. The difference between groups was analyzed by the log-rank test (*n* = 144 per group, *** *p* < 0.001). (**D**) Morphological features of resistant and susceptible larvae after 14 days of rearing are shown. (**E**) Representative Sanger sequencing traces of the SNP locus KZ288479.1_95621 for the resistant (R) and susceptible (S) groups. The box indicates the position of the single-nucleotide polymorphism. (**F**) Relative expression levels of the CSBV genome were quantified by qPCR in the NI, R, and S groups (*n* = 6 per group). Data are presented as mean ± SEM. Statistical significance was determined by one-way ANOVA followed by Tukey’s post hoc test. ns, not significant; *** *p* < 0.001.

Through laboratory rearing and subsequent CSBV inoculation, we observed a distinct dichotomy in disease manifestation among the larvae on day 7 post-inoculation (Figure 1A). The susceptible larvae exhibited a characteristic sac-like and translucent appearance, which is indicative of a high level of CSBV infection (Figure 1B). In contrast, the resistant larvae developed normally and were morphologically indistinguishable from the healthy larvae in the PBS control group.

We continued to monitor the developmental progress and survival of all larvae. Kaplan–Meier survival analysis revealed a statistically significant reduction in survival rates for the experimental group compared to the control group (Figure 1C). Further longitudinal observation until day 14 (the pupal stage) underscored the critical impact of this phenotypic dichotomy (Figure 1D). All larvae initially categorized as susceptible failed to pupate, succumbing to the disease. Conversely, every larva identified as resistant successfully underwent pupation, mirroring the developmental trajectory of the control larvae.

To genetically corroborate the phenotypic classification, we performed genotyping analysis for previously reported single nucleotide polymorphisms (SNP: KZ288479.1_95621) associated with CSBV resistance [32]. The genotyping results revealed a clear association between specific genotypes and the observed phenotypes. At SNP KZ288479.1_95621, individuals with the homozygous CC genotype were predominantly classified as resistant (R), while the heterozygote CT genotype was exclusively found in the susceptible (S) group (Figure 1E, Supplementary Table S1). This genotype–phenotype correlation confirms that the observed resistance and susceptibility are strongly linked to these specific genetic loci.

To unequivocally confirm that the observed phenotypes and mortality were due to CSBV infection, we performed reverse transcription polymerase chain reaction (RT-PCR) analysis on five randomly selected larvae from the virus-inoculated group. The resulting electrophoretic gel showed a distinct band at approximately 157 base pairs for all five samples (Figure S1), and subsequent sequencing verified the amplified fragments as the target CSBV genomic sequence. This provides direct molecular evidence that both resistant and susceptible phenotypes were successfully infected.

We next compared the viral load across groups by integrating the natural infection background. The Natural Infection group (NI, comprising the 40 qPCR-positive control individuals) exhibited a viral load that was not significantly different from that of the R group. In contrast, the S group showed a significantly higher relative viral load compared to both the NI and the R group (*** *p* < 0.001; Figure 1F).

### 3.2. Metabolic Profiling Reveals Distinct Metabolic Responses Between Susceptible and Resistant Larvae to CSBV Infection

To investigate the metabolic basis of susceptibility and resistance during CSBV infection, a non-targeted metabolomics analysis was performed on the control group, S group, and R group. Principal component analysis (PCA) clearly demonstrated significant differences in metabolic states among the three groups.

**Figure 2:**
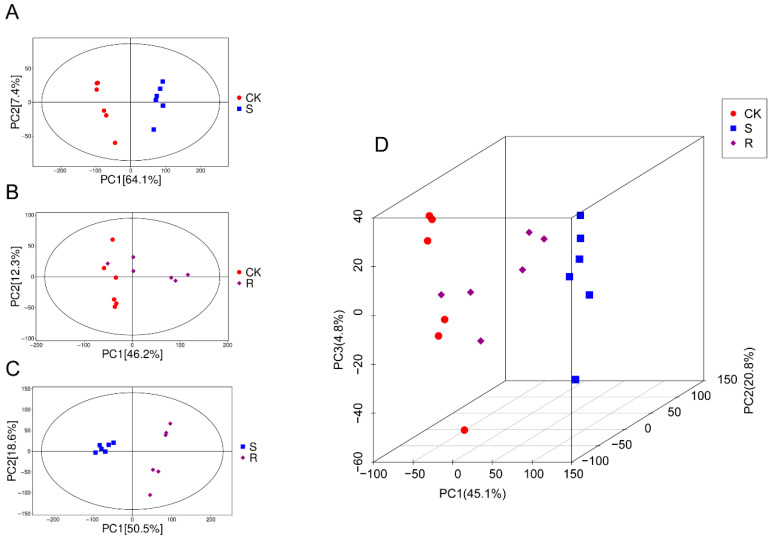
Non-targeted metabolomics analysis of larval gut. (**A**) Principal component analysis (PCA) plot between groups CK and S. (**B**) PCA plot between groups CK and R. (**C**) PCA plot between groups S and R. (**D**) Three-dimensional PCA plot of groups CK, S, and R.

First, in the comparison between the control group (CK) and the S group, the PCA score plot showed complete separation between the two groups (Figure 2A). The first principal component (PC1), accounting for 64.1% of the total variance, served as the primary dimension distinguishing the healthy and susceptible-infected states. The tight clustering of intra-group replicates indicated high experimental reliability. Second, the PCA model comparing the CK and R groups revealed a different pattern (Figure 2B). Despite good intra-group reproducibility, the CK and R groups did not separate, showing partial overlap. Third, a direct comparison between the S and R groups revealed the most pronounced separation (Figure 2C). The two groups formed distinct, non-overlapping clusters. PC1 (50.5%) and PC2 (18.6%) together explained 69.1% of the total variance. Finally, a three-dimensional PCA model incorporating all three groups (CK, S, and R) provided a comprehensive view of the post-infection metabolic landscape (Figure 2D). The S group formed an isolated cluster distant from the other two groups, while the R group was positioned between the CK and S groups, closer to and partially overlapping with the CK group.

Collectively, these PCA results demonstrated that susceptible larvae undergo a severe metabolic reprogramming, whereas the resistant larvae maintain a metabolic state largely similar to that of healthy controls.

### 3.3. Screening of Differential Metabolites

**Figure 3:**
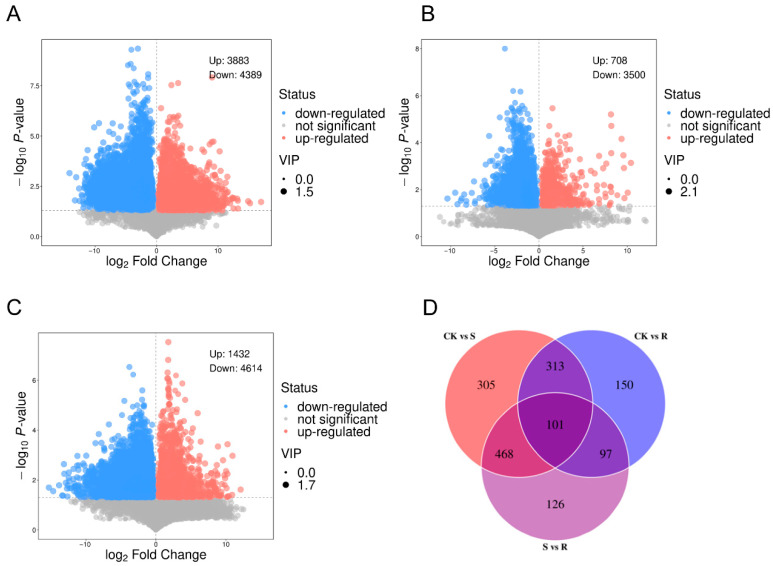
Differentially expressed metabolites (DEMs). (**A**) Volcano plot visualizing DEMs between the CK and the S group. The plot displays the statistical significance (−log10 (*p*-value)) versus the magnitude of change (log2 (fold change)). Metabolites with a significant increase in group S are highlighted in red, those with a significant decrease are highlighted in blue, and non-significant metabolites are shown in gray. The thresholds for significance were set at |log2(fold change)| > 1 and *p*-value < 0.05. (**B**) Volcano plot of DEMs between the CK and the R group. The same thresholds and color scheme as in (**A**) were applied to identify metabolites that were significantly upregulated or downregulated in group R compared to CK. (**C**) Volcano plot of DEMs from a direct comparison between the S and the R. This comparison highlights metabolic differences that are potentially associated with the differential survival outcome following viral challenge. (**D**) Venn diagram illustrating the unique and shared DEMs across the three groups (CK, S, and R). The diagram identifies core metabolic responses common to all CSBV-challenged larvae (S and R) versus the control (CK), as well as metabolites uniquely altered in susceptibility or resistance.

Volcano plot analysis revealed distinct metabolite profiles among the experimental groups (Figure 3A–C). The comparison between the CK and S groups identified 8272 differential metabolites, with 3883 upregulated in the S group and 4389 downregulated in the S group relative to the CK group. In contrast, the CK vs. R comparison showed 4208 differential metabolites, with 708 upregulated in the R group and 3500 downregulated in the R group relative to the CK group, indicating a more subdued metabolic response in resistant larvae. The direct S vs. R comparison demonstrated 6046 differential metabolites, with 1432 upregulated in the R group and 4614 downregulated in the R group relative to the S group, confirming fundamentally distinct metabolic states.

Venn diagram analysis identified 101 common differential metabolites across all comparisons (Figure 3D). Group-specific metabolites included 305 unique to CK vs. S, 150 unique to CK vs. R, and 126 unique to S vs. R comparisons, suggesting their potential roles in defining susceptibility- or resistance-specific metabolic pathways.

### 3.4. Characterization of Differential Metabolite Expression and Classification

**Figure 4:**
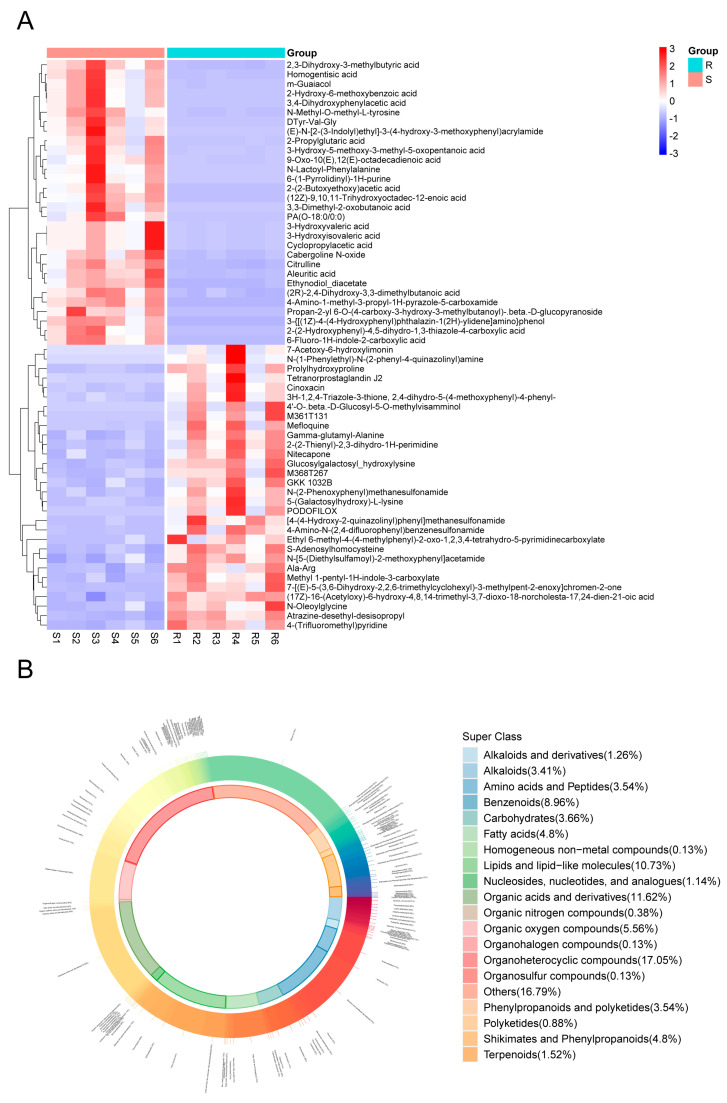
Differentially expressed metabolites (DEMs) between S and R groups. (**A**) Heatmap visualization of the 60 key DEMs selected to characterize groups S and R. The metabolites were chosen by taking the top 30 VIP metabolites from each group. Each row represents a metabolite, and each column represents a sample. The relative abundance of each metabolite is normalized by Z-score and indicated by color, with red representing high expression and blue representing low expression. (**B**) Donut chart showing the classification and proportional distribution of the DEMs between groups S and R.

A heatmap analysis of differential metabolites between S and R groups revealed distinct expression patterns (Figure 4A). The heatmap was generated by selecting the top 30 upregulated metabolites in the S group and the top 30 upregulated metabolites in the R group based on their VIP (variable importance in projection) scores. This allowed for a comprehensive comparison of the most significant metabolic shifts between the two groups. Metabolites significantly upregulated in the S group relative to the R group included 4-amino-1-methyl-3-propyl-1h-pyrazole-5-carboxamide, cyclopropylacetic acid, citrulline, and multiple hydroxy/hydroxyphenyl acids. Conversely, metabolites showing higher abundance in the R group encompassed various sulfonamides, s-adenosylhomocysteine, prolylhydroxyproline, gamma-glutamyl-alanine, and several lipid mediators including tetranorprostaglandin j2 and n-oleoylglycine.

Classification analysis of all identified metabolites demonstrated that organoheterocyclic compounds (18.37%), lipids and lipid-like molecules (10.34%), and organic acids and derivatives (11.67%) constituted the three most abundant categories (Figure 4B), suggesting their potential significance in CSBV infection response mechanisms.

### 3.5. Metabolic Pathway and Network Analysis

**Figure 5:**
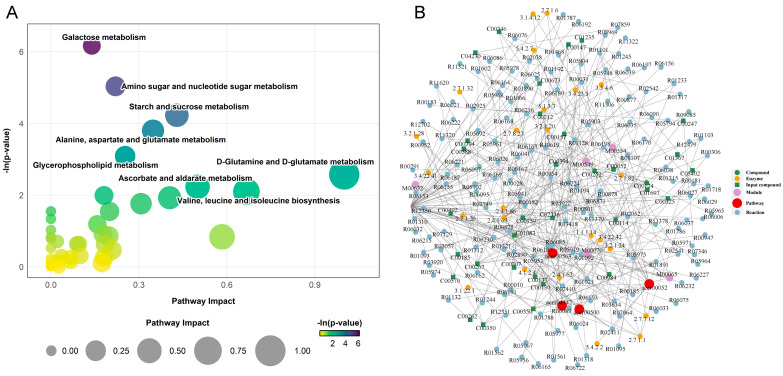
Pathway and regulatory network of differentially expressed metabolites (DEMs) between S and R groups. (**A**) Bubble plot presenting the results of the metabolic pathway analysis. Each bubble represents a metabolic pathway. The *x*-axis position and the size of the bubble correspond to the pathway impact value from topological analysis, with a larger size indicating a greater impact. The *y*-axis position and the color intensity of the bubble correspond to the −ln(*p*-value) from the enrichment analysis, with a darker color indicating a smaller *p*-value and a higher degree of statistical significance. (**B**) Regulatory interaction network analysis based on KEGG pathway enrichment. In the network plot, nodes are represented as follows: red dots denote a metabolic pathway; yellow dots represent enzyme information associated with a metabolite; green dots indicate background compounds of a metabolic pathway; purple dots represent a molecular module of compounds; blue dots indicate a chemical interaction reaction; and green squares represent the differentially expressed metabolites identified in this comparison.

To systematically elucidate the functional implications of the differential metabolites between S and R larvae, pathway enrichment analysis was performed using MetaboAnalyst 6.0. The analysis revealed five metabolic pathways that were significantly altered (*p* < 0.05), painting a clear picture of divergent metabolic strategies (Figure 5A).

Most differential metabolites were significantly upregulated in the S group. This widespread metabolic activation was prominently featured in pathways related to carbohydrate metabolism and energy production. Specifically, we observed substantial upregulation in galactose metabolism (e.g., glucose, udp-galactose), amino sugar and nucleotide sugar metabolism (e.g., n-acetylglucosamine, udp-glucose), and starch and sucrose metabolism (e.g., trehalose, fructose). Notably, key metabolites such as glucose and udp-glucose were hubs, being enriched across multiple pathways. Furthermore, intermediates of the alanine, aspartate, and glutamate metabolism pathway (e.g., glutamate, α-ketoglutaric acid, and pyruvate) were also accumulated in the S group.

In stark contrast, the R group exhibited a highly targeted metabolic response. Out of all significantly altered metabolites across the five key pathways, only four were specifically upregulated in the R group. These included udp-n-acetylglucosamine (in amino sugar metabolism), l-asparagine (in alanine, aspartate, and glutamate metabolism), and two critical intermediates in glycerophospholipid metabolism: sn-glycerol 3-phosphate and phosphorylcholine. This stark dichotomy indicates that susceptibility is characterized by a generalized, and potentially dysregulated, metabolic activation, whereas resistance is associated with a precise upregulation of metabolites involved in structural integrity and membrane biosynthesis.

Furthermore, construction of a comprehensive regulatory network revealed the intricate molecular interactions underlying these pathway perturbations (Figure 5B). The network analysis identified key functional modules, including nucleotide sugar biosynthesis (M00549, M00554) and gpi-anchor biosynthesis (M00065), as central hubs connecting the metabolic alterations. Critical enzymatic nodes such as hexokinase (2.7.1.1), galactokinase (2.7.1.6), and choline kinase (2.7.1.32) served as major regulators within this network, coordinating the flow of metabolites through the disrupted pathways. The network topology demonstrated that the differential metabolites formed dense interconnections through various enzymatic reactions and biochemical transformations, particularly around sugar metabolism and phospholipid biosynthesis pathways.

### 3.6. Resistant Larvae Exhibit Enhanced Antioxidant Enzyme Activity in Gut Tissue Following CSBV Infection

**Figure 6:**
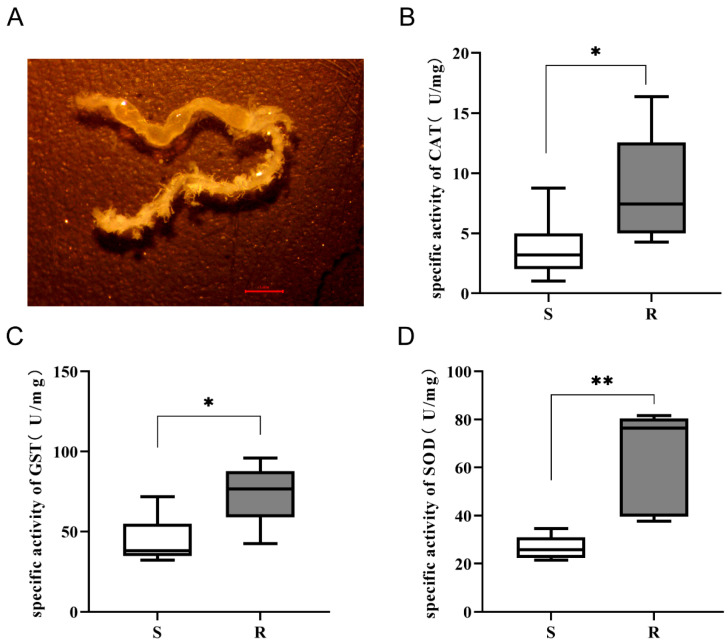
Gut antioxidant enzyme activities in *A. cerana* larvae. (**A**) The gut of *A. cerana* larva. (**B**–**D**) Activities of catalase (CAT) (**B**), glutathione S-transferase (GST) (**C**), and superoxide dismutase (SOD) (D) in the guts of S and R groups. Data are presented as mean ± SD, and differences were analyzed by Student’s *t*-test. (*n* = 6 per group, * *p* < 0.05, ** *p* < 0.01).

To validate the oxidative stress implications suggested by our metabolomic profiling (e.g., the accumulation of oxidative stress markers like DOPAC in susceptible larvae), we investigated the antioxidant defense capacity in the gut tissue of CSBV-infected larvae (Figure 6A). The activities of three key antioxidant enzymes—CAT, GST, and SOD—were measured and compared between the S and R groups.

The results demonstrated a consistently and significantly stronger antioxidant response in the resistant larvae. The specific activities of all three enzymes were markedly higher in the R group compared to the S group. The mean CAT activity in resistant larvae was approximately 2.5-fold higher than in susceptible larvae (Figure 6B). Similarly, the mean activities of GST and SOD were 1.5-fold and 2.0-fold higher in the R group, respectively. Statistical analysis confirmed these differences were significant (Figure 6C,D).

## 4. Discussion

This study provided a comprehensive analysis of the metabolic and physiological mechanisms underlying resistance and susceptibility to CSBV in *A. cerana* larvae. By integrating controlled viral inoculation, untargeted metabolomics, and biochemical validation, we showed that the resistant phenotype is not merely the absence of disease symptoms, but an active, protective state characterized by a distinct metabolic profile and a robust antioxidant defense system, which allows larvae to survive and develop despite viral presence [22].

Our qPCR analysis elucidated a clear correlation between CSBV viral load and phenotypic outcomes. The susceptible (S) group exhibited significantly elevated viral titers, directly linking high viral replication to symptomatic disease. The most critical finding lies in the comparison between the NI and R groups. We observed no significant difference in CSBV expression levels between them. This result, combined with our survey revealing an 83% CSBV infection rate in the Control group (consistent with the high subclinical prevalence of ~90% reported by Hassanyar et al. [37], is highly informative. It demonstrates that the resistant (R) phenotype is characterized not by the absence of infection, but by the ability to suppress viral replication to a basal, asymptomatic level—indistinguishable from that of naturally infected controls. This mirrors findings in honey bees infected with Deformed Wing Virus (DWV), where asymptomatic individuals maintain low viral titers despite widespread infection, often through active immune containment [38].

The emergence of distinct R and S phenotypes from a single queen can be primarily explained by the polyandrous mating system of honey bees. As a queen mates with multiple drones, her offspring are a cohort of half-siblings comprising multiple patrilines [39]. This genetic diversity is a key substrate for differential pathogen responses, as established evidence shows that genetic diversity within a colony reduces disease prevalence and that different patrilines vary significantly in their pathogen resistance [40]. Our genotyping analysis for the SNP locus KZ288479.1_95621, a C > T variant previously associated with resistance to CSBV, directly supports this mechanism [32]. Specifically, the resistant C allele was found to be distributed differentially across patrilines, providing a clear molecular basis for the variation in CSBV resistance observed among the queen’s offspring. Thus, the genetic architecture created by polyandry is a fundamental mechanism driving the observed divergence in survival and metabolic profiles.

Our PCA results revealed a clear differentiation between the S and control groups, suggesting that CSBV infection triggers profound and systemic metabolic reprogramming in susceptible individuals. This is consistent with previous studies of host–virus interactions, where viral infection is often associated with a “metabolic takeover,” which enables viral replication and pathogenesis [41,42]. In contrast, the partial overlap between the R and control groups indicated that resistance is maintained by a mechanism that buffers metabolic dysregulation, preventing the pathological shifts seen in the S larvae. This suggests that virus resistance in the R group operates through mechanisms that preserve metabolic homeostasis, similar to “disease tolerance” mechanisms observed in other organisms [43]. The marked separation between the S and R groups confirms that these are two distinct biological outcomes: one leads to survival and homeostasis, while the other results in pathological dysregulation and death.

The scale of metabolic disruption in the S group is striking, with over 8200 differential metabolites compared to ~4200 in the R group. This highlights the severe metabolic burden imposed by viral infection on susceptible larvae. The downregulation of many metabolites in the R group suggests a strategy of metabolic conservation, possibly through suppression of non-essential physiological processes, to prioritize immune defense and virus resistance. This adaptive strategy is similar to the metabolic reprogramming observed in other organisms under biotic stress [44,45].

Our pathway analysis goes beyond listing altered metabolites to reveal functional modules that are specifically disrupted by CSBV infection. The significant enrichment of pathways related to galactose, starch, and sucrose metabolism in the S group suggests severe disruptions in energy harvesting and allocation, which are characteristic of viral-induced metabolic crises [46,47]. Viruses reprogram host metabolism, hijacking cellular resources to meet the biosynthetic demands of their replication [48]. Indeed, the S group exhibited upregulation in carbohydrate and nucleotide sugar metabolism, which could be interpreted as “metabolic hijacking,” where the virus co-opts host resources for replication, as evidenced by the increased production of metabolites like udp-glucose, crucial for viral biosynthesis [49]. Concurrent upregulation of energy cycle intermediates indicates a futile, dysregulated stress response. In stark contrast, R larvae displayed a precise “targeted defense” strategy. The specific upregulation of udp-n-acetylglucosamine points to the reinforcement of the chitinous cuticle and peritrophic matrix, a critical physical barrier in insect innate immunity [50]. Additionally, upregulation of energy cycle intermediates signals a dysregulated stress response in the S group. In contrast, the R group exhibited a more focused “targeted defense” strategy. Specifically, the upregulation of udp-n-acetylglucosamine points to the reinforcement of the chitinous cuticle and peritrophic matrix—key components of insect innate immunity [45]. Moreover, disturbances in glycerophospholipid metabolism and gpi-anchor biosynthesis in the S group suggest compromised gut epithelial integrity, potentially facilitating viral spread and impairing nutrient absorption [51].

Viral infection causes mitochondrial dysfunction and ROS production, which facilitates viral replication [52]. Our results establish a critical link between host antioxidant capacity and viral pathogenesis. The R group exhibited a significant increase in γ-glutamyl-alanine, a key intermediate in GSH biosynthesis [53], along with elevated antioxidant enzyme activity (Figure 6), indicating enhanced antioxidant defense. We propose that this potentiated response in the R group effectively scavenges virus-induced ROS, thereby disrupting the oxidative environment necessary for viral replication [54]. This suppression of ROS-mediated proviral signaling likely contributes to the group’s antiviral phenotype and survival. In contrast, the S group showed reduced antioxidant enzyme activity and lower γ-glutamyl-alanine levels, indicating a weakened antioxidant defense. This likely results in elevated intracellular ROS and enhanced inflammatory signaling, creating an oxidative environment conducive to viral replication and spread [55]. The failure to reestablish redox homeostasis ultimately leads to severe phenotypic outcomes and mortality, highlighting the critical role of host antioxidant capacity—particularly the glutathione synthesis pathway [54]—in shaping infection outcomes through modulation of the intracellular redox state.

The accumulation of s-adenosylhomocysteine (SAH) in resistant larvae presents a fascinating potential antiviral mechanism. SAH is a potent feedback inhibitor of cellular methyltransferases [56]. Many viruses, including those with positive-sense RNA genomes like CSBV [10,11,12], depend on the host’s methylation machinery for capping their RNA to ensure efficient translation and evade immune detection [57,58]. Elevated SAH in R larvae could therefore limit viral replication by inhibiting methylation processes, offering a sophisticated form of “metabolic immunity” against the virus [59].

Conversely, the accumulation of DOPAC and Homogentisic acid in susceptible larvae serves as a stark biomarker of pathology. DOPAC is a primary metabolite of dopamine [60], and its elevation is a well-established indicator of intense oxidative stress [61]. Homogentisic acid accumulation typically signifies a blockage in the tyrosine catabolism pathway, reflecting a broader state of metabolic dysfunction and loss of homeostasis [62,63]. Furthermore, the accumulation of various hydroxy/hydroxyphenyl acids in S larvae suggests potential dysbiosis of the gut microbiota or impaired host–microbe co-metabolism, which is critical for insect health and immunity [64,65]. This aligns with the observed gut tissue damage and failure of nutrient assimilation in susceptible individuals.

The most compelling validation of our metabolomic findings comes from the direct measurement of antioxidant enzyme activities. The metabolomic data, particularly the elevated DOPAC in S larvae, suggested severe oxidative stress, and the physiological assay confirmed it unequivocally. The coordinated upregulation of CAT, GST, and SOD in the gut of resistant larvae demonstrates a highly effective countermeasure against this threat, preserving cellular integrity [66,67].

The resistant phenotype, however, appears to extend beyond just antioxidant capacity. The higher levels of n-oleoylglycine in R larvae suggest the engagement of specialized pro-resolving mediators. Although its function in insects is less defined, in mammalian systems, n-acyl glycines like n-oleoylglycine are known to possess anti-inflammatory and pro-resolving properties, actively limiting excessive inflammation and promoting tissue homeostasis [68,69,70]. Similarly, the elevation of the collagen-derived dipeptide prolylhydroxyproline in R larvae may indicate an enhanced capacity for tissue repair and extracellular matrix remodeling [71,72], allowing for the swift healing of virus-induced damage.

A significantly higher accumulation of citrulline in the intestines of susceptible (S) larvae compared to their resistant (R) counterparts. This distinct metabolic signature suggests a dysregulated nitrogen oxide (NO) synthesis pathway in S larvae [73]. In insects, NO is a critical immune effector whose production must be tightly controlled [74]; low levels reversibly modulate respiration via COX inhibition, while excessive, uncontrolled generation leads to irreversible inhibition of respiratory complexes, causing mitochondrial dysfunction, oxidative stress, and cell death [73,75]. The elevated citrulline in S larvae may, therefore, indicate a failed or overly active immune response that results in detrimental overproduction of NO. This scenario is further exacerbated by the concurrently observed deficient antioxidant response in S larvae (Figure 6). We propose that this synergy of excessive NO-related oxidative stress and deficient antioxidant defense inflicts mitochondrial and cellular damage, driving the severe pathology in susceptible larvae. In contrast, the lower citrulline in R larvae reflects a more stringent regulatory mechanism, likely contributing to their resistance by preventing such immunopathological damage.

While this study provides valuable insights into the metabolic basis of CSBV resistance through a comprehensive metabolomic perspective, certain limitations should be acknowledged. The correlative nature of our metabolomics data, while highly suggestive, requires future functional validation (e.g., through genetic or pharmacological interventions) to establish causality for the role of key metabolic pathways in CSBV resistance. Furthermore, the analysis of whole gut tissue may average critical, cell-type-specific metabolic responses, a common challenge in bulk tissue analysis that could be resolved by future single-cell approaches [76,77].

In conclusion, this study demonstrates that survival to CSBV infection in *A. cerana* larvae is not a passive event, but rather the result of an active metabolic and physiological defense. The distinct metabolic profiles observed in the R and S groups provide new insights into the mechanisms of viral resistance and susceptibility. These findings deepen our understanding of host–pathogen interactions and offer potential metabolic biomarkers for breeding programs aimed at enhancing viral resistance in *A. cerana* populations.

## Data Availability

Data sharing is not applicable to this article as no datasets were generated or analyzed during the current study.

## Supplementary Materials

The following supporting information can be downloaded at: https://www.mdpi.com/article/10.3390/insects16121283/s1, Figure S1: Gel electrophoresis of PCR detection for CSBV; Table S1: Allele frequencies (C/T) for SNP KZ288479.1_95621 in R and S groups.
