# Supplementary material for: Larval Metabolic and Physiological Mechanisms Underlying Resistance to Chinese Sacbrood Virus in Apis cerana
Source: Insects. 2025 Dec 18;16(12):1283. doi: 10.3390/insects16121283 (PMC12734291; doi:10.3390/insects16121283)
Supplement: Supplementary file 1 [file insects-16-01283-s001.zip › insects-4013723-supplementary.pdf]

Table S1 Allele frequencies (C/T) for SNP KZ288479.1_95621 in R and S groups.

Group	Sample Size	Frequency of Genotypes			Frequency of Alleles c				
		CC	CT	TT	C	P_C[a]	T	P_T[a]	p-value (χ^2 test)
R	12	9	3	0	21	0.875	3	0.125	0.009
S	12	1	11	0	13	0.542	11	0.458	

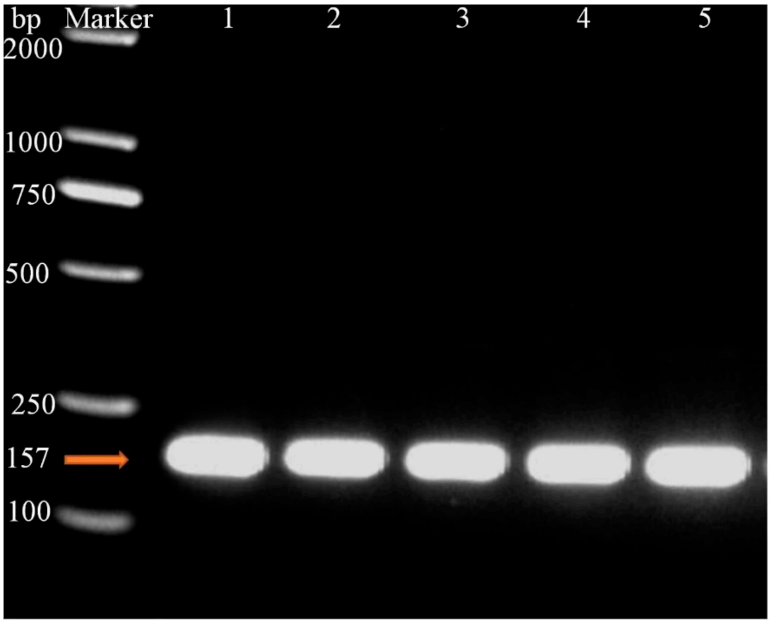

Figure S1 Gel electrophoresis of PCR detection for CSBV in randomly selected larvae. The expected CSBV fragment size is 157 bp.
